# Living donor domino liver transplantation in a hepatitis C virus/human immunodeficiency virus-coinfected hemophilia patient: a case report

**DOI:** 10.1186/s40792-020-00944-4

**Published:** 2020-07-29

**Authors:** Hidekazu Yamamoto, Yasuhiko Sugawara, Yuzuru Sambommatsu, Keita Shimata, Daiki Yoshii, Kaori Isono, Masaki Honda, Taro Yamashita, Shuzo Matsushita, Yukihiro Inomata, Taizo Hibi

**Affiliations:** 1 Department of Pediatric Surgery and Transplantation, Kumamoto University Graduate School of Medical Sciences, 1-1-1, Honjo, Chuo-ku, Kumamoto, 860-8556 Japan; 2 Department of Neurology, Kumamoto University Graduate School of Medical Sciences, Kumamoto, 860-8556 Japan; 3 Center for AIDS Research, Kumamoto University, Kumamoto, 860-8556 Japan; 4 Department of Surgery, Kumamoto Rosai Hospital, Kumamoto, 866-8533 Japan

**Keywords:** Direct-acting antivirals, HCV/HIV-coinfection, Liver transplantation, Living donor domino liver transplantation, Sofosbuvir/ledipasvir

## Abstract

**Background:**

Outcome of the liver transplantation (LT) is worse in hepatitis C virus (HCV)/human immunodeficiency virus (HIV)-coinfected patients compared to patients infected with HCV alone. We report the world’s first case of living donor domino liver transplantation (LDDLT) using a familial amyloid polyneuropathy (FAP) liver in a coinfected recipient with HCV-related liver cirrhosis.

**Case presentation:**

The recipient was a 43-year-old male with a CD4 cell count of 52/μL and undetectable HIV-RNA at the time of LT. He received a domino liver graft from a 41-year-old female with FAP. No acute cellular rejection or infection occurred after LT. HCV recurrence was confirmed histologically on the posttransplant day 34. Peginterferon/ribavirin therapy resulted in non-response; however, the patient achieved a sustained viral response with sofosbuvir (SOF)/ledipasvir (LDV). Currently, HCV and HIV testing are negative, and symptomatic de novo amyloidosis has not occurred.

**Conclusions:**

LDDLT allows successful LT in HCV/HIV-coinfected patients; posttransplant HCV recurrence can be successfully treated with anti-viral therapy.

## Background

The introduction of anti-retroviral therapy (ART) has improved outcome in patients infected with the human immunodeficiency virus (HIV) [1, 2]. In the ART era, end-stage liver disease related to hepatitis C virus (HCV) and hepatitis B virus (HBV) coinfection has emerged as a main cause of morbidity and mortality in HIV-infected individuals; HCV- and HBV-related cirrhosis have become the most common indication for liver transplantation (LT) among the HIV patients.

Domino liver transplantation (DLT) has been established as a tool that contributes to expansion of the donor pool. Except for the production of an abnormal protein or enzyme, these livers are morphologically normal and fully functional. However, the metabolic disease of the donor is usually transmitted to the DLT recipient several years after LT [3–5]. Patients with familial amyloid polyneuropathy (FAP) are the most common donor in DLT. In most studies of long-term outcome in DLT, FAP patients received livers from deceased donors [6–10]. Therefore the data regarding living donor domino liver transplantation (LDDLT) is limited [11, 12]. We report the first case of successful LDDLT in a HCV/HIV-coinfected patient

## Case presentation

The patient was a 43-year-old male with HCV-related end stage liver disease (ESLD) coinfected with HIV. He was diagnosed with hemophilia B at the age of 6 years and later infected with both HCV and HIV by transfusion with contaminated plasma-derived factor concentrates. Initially, he was a non-progressor for many years after HIV infection was diagnosed. However, at age 35 years, he began ART. Eight years later, his CD4 count recovery was attenuated, and the ART was changed from efavirenz to raltegravir. Subsequently, the patient developed ART-induced liver toxicity and ART was discontinued. His liver function continued to worsen due to HCV-related ESLD and became life-threatening. At this point, the patient was evaluated for liver transplantation: Laboratory tests revealed serum total bilirubin (T-Bil) 25.4 mg/dL, aspartate aminotransferase (AST) 57 U/L, alanine aminotransferase (ALT) 39 U/L, prothrombin time-international normalized ratio (PT-INR) 1.64, activated partial thromboplastin time (APTT) 66 s, platelet count 84 × 10^3^/μL, and factor IX 6% of standard. The inhibitor of factor IX did not develop. The HCV-RNA viral load was 5.5 log IU/mL and genotype was 1a. Model for end-stage liver disease (MELD) score was 24, Child-Pugh score was 10, CD4 cell count was 52/μL (CD4/CD8 ratio 0.09), and HIV-RNA was undetectable.

He received a domino liver graft from a 41-year-old female with FAP. The whole liver without inferior vena cava was transplanted using the piggy-back technique. The right hepatic vein of the liver graft was anastomosed to the stump of recipient’s right hepatic vein. The unified middle and left hepatic vein of the liver graft was anastomosed to the recipient’s trunk of the middle and left hepatic vein in an end-to-end fashion. The portal vein was anastomosed with the branch patch (left and right branches of the recipient’s portal vein) in an end-to-end fashion. After reperfusion, the hepatic artery was microsurgically reconstructed. Duct-to-duct anastomosis was employed for the biliary reconstruction. Additionally, splenectomy was performed for prevention of interferon-induced thrombocytopenia in the posttransplant treatment of hepatitis C. The length of operation was 11 h 30 min. The blood loss was 2936 mL and a total of 569 mL of red cells concentrates, and 720 ml of fresh-frozen plasma was infused during transplantation. Histopathology of the explanted liver showed the findings compatible with HCV liver cirrhosis (necroinflammatory activity score, A3; fibrosis score, F4).

One thousand U of recombinant factor was infused every 4–5 days before transplantation. Immediately before transplantation, 5000 U of recombinant factor was given by bolus infusion, and recombinant factor was administered continuous and additional eight bolus infusion in total to keep APTT less than 45 s intraoperatively. After transplantation, recombinant factor was given continuously until 2 days.

**Fig. 1:**
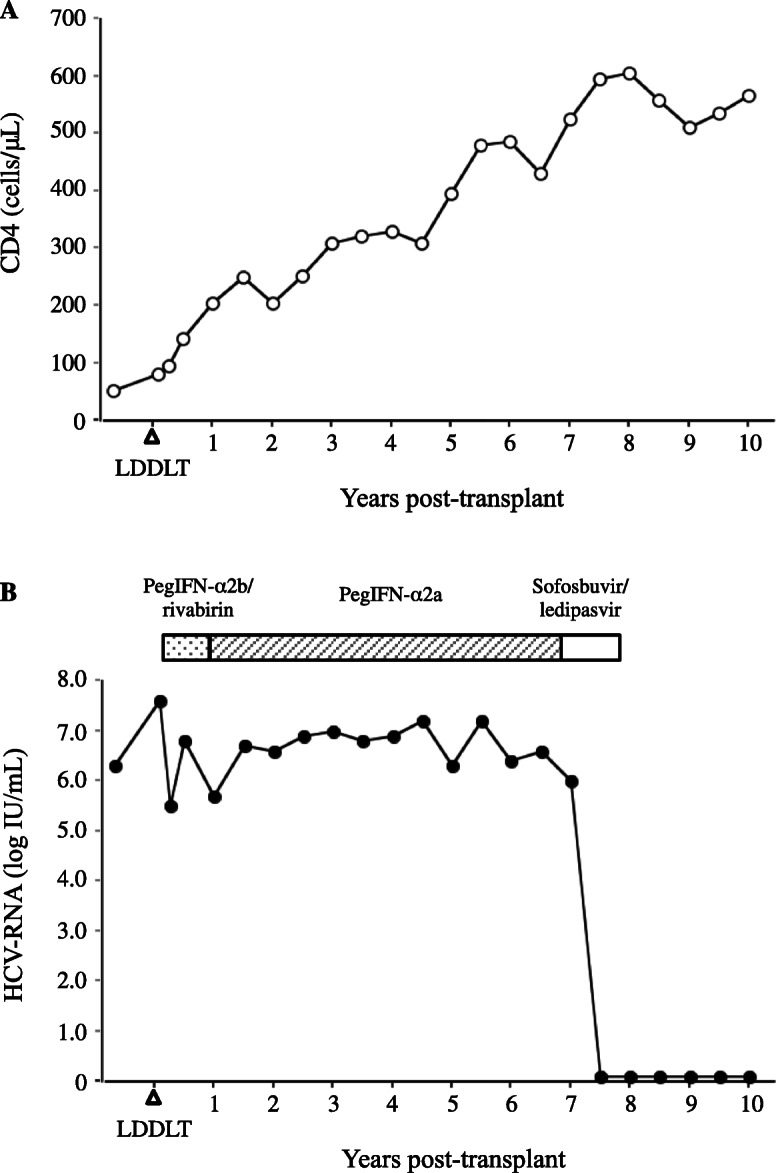
**a** CD4 cell count following liver transplantation. LDDLT, living donor domino liver transplantation. **b** HCV-RNA titers following liver transplantation. Abbreviation: *LDDLT*, living donor domino liver transplantation

Postoperatively, the patient received tacrolimus and steroids for immunosuppression; the steroids were rapidly tapered within 3 months after LT. The target trough levels of tacrolimus were 10 to 15 ng/mL through the week 2 postoperatively, and then around 10 ng/mL until 1 month postoperatively, 5 to 10 ng/mL until 3 months. The patient has remained free of both infection and acute cellular rejection since LT and liver function testing has been stable. ART consisting of emtricitabine, tenofovir, and raltegravir was reinitiated on the posttransplant day 1. Four months after LT, the CD4 cell count gradually increased and exceeded more than 100/μL (Fig. 1a). Darunavir and ritonavir were then added, and his CD4 cell count reached 204/μL 12 months after LT. The HIV viral load was persistently negative. In terms of immunosuppression, trough level of tacrolimus was around 7.0 ng/mL by 2.0 mg twice daily before the induction of darunavir. After the induction of darunavir, tacrolimus was then given as a single dose of 0.05 mg every 4–7 days due to maintaining trough level of 6.0–8.0 ng/mL.

HCV recurrence was diagnosed histologically (necroinflammatory activity score, A1; fibrosis score, F0-1) on the posttransplant day 34. He then received antiviral therapy consisting of peginterferon-α2b and ribavirin on the posttransplant day 41. At the start of therapy, the HCV-RNA viral load was 7.5 log IU/mL. However, he had no response, so was changed to peginterferon-α2a, which he received for 6 years; HCV-RNA titers remained stable. Seven years after LT, liver biopsy showed stage F3 fibrosis and laboratory analysis revealed AST 49 U/L, ALT 32 U/L, T-Bil 0.4 mg/dL, and creatinine 0.96 mg/dL. His Child-Pugh score was 6. Peginterferon-α2a was converted to sofosbuvir (SOF)/ledipasvir (LDV) (direct-acting antivirals (DAAs) were approved to be covered by health insurance in Japan at that time). At the start of SOF/LDV therapy, the HCV-RNA viral load was 6.0 log IU/mL; serum HCV-RNA became undetectable 14 days after initiation of SOF/LDV. Since then, the patient has maintained a sustained viral response (SVR), and HCV-RNA has remained undetectable (Fig. 1b). After the initiation of SOF/LDV therapy, transaminase was normalized and renal function kept normal.

Currently, both HCV and HIV viral loads are negative, and the patient is well at 11 years after LT. Symptomatic de novo amyloidosis has not yet occurred, although faint amyloid deposition was observed in stomach and skin biopsies at 8 and 10 years after LDDLT, respectively.

This study was approved by the ethics committee of Kumamoto University Hospital (No. 892).

## Discussion

Liver cirrhosis due to HCV/HIV-coinfection is a remarkable indication for LT. In addition, HCV/HIV-coinfected patients affected by portal hypertension when cirrhosis is absent may be considered for LT as well [13–15]. In the ART era, LT for HIV-infected patients with liver disease not related with HCV had an excellent outcome [16, 17]. However, the outcome of LT is worse in HCV/HIV-coinfected patients than patients infected with HCV alone due to rapid progression of fibrosis and a higher incidence of severe recurrent HCV infection [18–20]. Therefore, it is important to consider the treatment of recurrent HCV after LT. One study found a significantly higher rate of progression to a fibrosis score ≥ F2 2 years after LT in HCV/HIV-coinfected patients (71%) compared to patients infected with HCV alone (40%) [18].

In previous reports, the interferon-based anti-HCV treatment for HCV/HIV-coinfected LT recipients was associated with SVR rates ranging from 11 to 27% [19, 21, 22]. In addition, the effectiveness of DAAs against HCV has recently been shown for both HCV/HIV-coinfected patients and patients infected by HCV alone. SOF-based antiviral therapy is highly effective after LT in HCV/HIV-coinfected recipients, with SVR rates for HCV recurrence ranging from 89 to 100% [23–26]. In the present case, peginterferon with rivabirin was not effective in preventing recurrent HCV after LT. However, 14 days after SOF/LDV therapy, HCV-RNA was undetectable, and our patient achieved a SVR.

Operative criteria for LT from the point of view of HIV infection in many centers are pretransplant CD4 cell counts > 100/μL or 200/μL and absence of HIV viremia [27–29]. However, Ragni et al. reported that the critical determinant for survival was posttransplant CD4 count rather than pretransplant CD4 count [30]. Although pretransplant CD4 cell count was lower in the present case than in previously reported ones, the postoperative outcome was uneventful without infectious complications. It may be important to appropriately control immunosuppressive therapy and routinely monitor for infection.

Liver transplantation for hemophilia has a greater risk of intra- and postoperative bleeding. Several reports have discussed the efficacy of the administration of recombinant factor by continuous and/or bolus infusion as a bridge until a newly transplanted liver begins to produce and maintain adequate factor IX activity [31–33]. In this case, we could control intra-operative hemostasis well by the combination of the continuous infusion and intermittent bolus infusion with close monitoring. Additionally, our patient was given recombinant factor until 2 days posttransplant.

We have previously reported that DLT using a whole liver from a living donor liver transplantation from a FAP patient presents satisfactory results [11]. The indications for DLT included hepatocellular carcinoma (HCC), age > 50 or 60 years, age > 40 years with hepatitis C cirrhosis, and late retransplantation [7, 10]. We believe that these were indications of DLT in case of a small number of DDLT in Japan compared with other countries. The priority was the urgency to save lives regardless of the age or the original disease of the patient. The next was one or more factors that had been set for ordinary DLT in other countries as HCC, > 50 or 60 years of age, high MELD score, and late retransplantation. In case of such a patient, urgent LT was recommended to save life, in addition to the co-infection of HCV/HIV that was considered to have poor prognosis compared with non-coinfected HCV. This was the reason why this patient was selected as the final candidate among the patients in the list for DDLT in our institution.

It is reported that several patients would develop the amyloidosis 3 years after DLT earliest [34]. Rodrigues et al. reported that 10 of 81 FAP liver recipients eventually required retransplantation due to de novo amyloidosis [35]. Fortunately, symptomatic de novo amyloidosis has not occurred in our patient in the 11 years since LT. Despite the risk of amyloidosis, we believe that DLT using FAP livers is a viable option given today’s shortage of liver donors.

## Conclusions

We report the world’s first case of LDDLT in a HCV/HIV-coinfected who was a long survivor. DLT with a FAP liver can be a treatment option for liver cirrhosis associated with HCV/HIV-coinfection, although de novo amyloidosis may later occur in the recipient. DAA therapy for recurrent HCV after LT is safe and effective in HCV/HIV-coinfected patients.

## Abbreviations

ART: Anti-retroviral therapy
HIV: Human immunodeficiency virus
HCV: Hepatitis C
HBV: Hepatitis B
LT: Liver transplantation
DLT: Domino liver transplantation
FAP: Familial amyloid polyneuropathy
LDDLT: Living donor domino liver transplantation
ESLD: End stage liver disease
T-Bil: Total bilirubin
AST: Aspartate aminotransferase
ALT: Alanine aminotransferase
PT: Prothrombin time-international normalized ratio
APTT: Activated partial thromboplastin time
MELD: Model for End-stage Liver Disease
SOF/LDV: Sofosbuvir/ledipasvir
DAAs: Direct-acting antivirals
SVR: Sustained viral response
HCC: Hepatocellular carcinoma
